# Changes in Urine Microalbumin-to-Creatinine Ratio in Children with Sickle Cell Disease over Time

**DOI:** 10.3389/fped.2016.00106

**Published:** 2016-10-07

**Authors:** Ibrahim F. Shatat, Suparna Qanungo, Shannon Hudson, Marilyn A. Laken, Susan M. Hailpern

**Affiliations:** ^1^Pediatric Nephrology and Hypertension, Sidra Medical and Research Center, Doha, Qatar; ^2^College of Nursing, Medical University of South Carolina, Charleston, SC, USA; ^3^Weill Cornell Medical College, New York, NY, USA; ^4^Independent Epidemiology Consultant, Saratoga, CA, USA

**Keywords:** microalbuminuria, progression, sickle cell nephropathy, children, young adults

## Abstract

**Background:**

Approximately 20% of children with sickle cell disease (SCD) have microalbuminuria (MA). Very little is known about the progression of MA in children and young adults with SCD.

**Methods:**

In this study, we analyzed 5-year EMR data of 373 children [with ≥2 microalbumin-to-creatinine (MA/Cr) ratio measurements] followed at the Medical University of South Carolina to determine the rate, direction, magnitude, and predictors of MA/Cr change over time.

**Results:**

Age range was 1–22 years; mean 10.2 ± 5.2 years, 49.5% were males. Median follow-up duration was 3.12 ± 1.16 years. At baseline, 328 children had normal (<20 mg/L) MA level. Forty-five (12.1%) of children had MA (≥20 mg/L), of which 91% were ≥8 years and 21 (47%) continued to have MA at the end of the study period. On the other hand, during the study period, 24 new patients developed MA and 24 normalized their MA to levels <20 mg/L. In multivariate logistic regression model, age and bilirubin levels were predictive of MA/Cr increase in patients who received at least one blood transfusion during the study period. Baseline MA level was not predictive of the change in MA/Cr.

**Conclusion:**

In children and young adults, microalbuminuria is considered a marker of early renal injury. Over time, MA/Cr levels may increase or decrease. Further studies are needed to confirm our findings, assess the reliability of MA as marker of long-term renal injury, and identify high risk patients with SCD likely to have worsening of MA over time.

## Introduction

Sickle cell disease (SCD) affects more than 100,000 African-Americans (AA) in the United States. The single gene mutation causes distortion of red blood cells, which impairs oxygen delivery to every organ in the body. The entire nephron is affected by endothelial dysfunction, vaso-occlusion, ischemia, infarction, and ultimately nephron loss. Evidence of renal disease begins early in childhood and often progresses to chronic kidney disease (CKD) in adults (1).

A mild to moderate increase of urinary albumin has a high prevalence in patients with diabetes mellitus, hypertension, and chronic kidney disease (2, 3). This so-called microalbuminuria appears to be a risk marker for renal and cardiovascular diseases later in life (4, 5).

The improved survival of patients with SCD has led to an increase in the incidence of nephropathy, along with other target-organ damage, which often causes end-stage renal failure (1, 6, 7). The average age of renal failure is 40 years, but early signs, such as microalbuminuria, may be apparent in childhood (1). In fact, the prevalence of microalbuminuria or proteinuria in children with SCD has been shown to range from 18 to 28%. Though it can be intermittent in an individual, there is a correlation between MA levels and increasing age and decreasing hemoglobin levels in children and adolescents with SCD (8–13). To our knowledge, this is the first large pediatric study which aims to study progression and predictors of urinary microalbumin-to-creatinine change over time.

## Patients and Methods

### Study Population and Variables

This is a retrospective analysis of data from the Medical University of South Carolina (MUSC) EMR for the years 2009–2014. Children with SCD followed at the MUSC pediatric sickle cell disease clinic (the largest program in the state of SC) were identified from the EMR using relevant ICD-9 codes (282.41, 282.42, 282.60, 282.61, 282.62, 282.63, 282.64, 282.68, and 282.69); duplicates were excluded by matching to medical record number before de-identification. Children and young adults 1–22 years of age with the diagnosis of sickle cell disease with at least two MA/Cr measurements were included in this study.

At MUSC, a protocol for urine MA/Cr screening was started in 2008 to collect midstream urine samples at routine follow-up visits at the Sickle cell clinic, measurements were avoided within 2 weeks of hospitalization, any form of vaso-occlusive crises, or febrile illnesses.

Three hundred seventy-three children and young adult unique patients met the above criteria. To assess for magnitude and direction of change (i.e., increase or decrease) in microalbuminuria levels over time, the ratio of urinary microalbumin to urinary creatinine (MA/Cr) in micrograms per milligram was calculated and utilized for all patients. For clinical practice, microalbuminuria (MA) was defined as Urine MA of ≥20 mg/L (14). In this manuscript, MA refers to the absolute measurement known to define clinical microalbuminuria, while MA/Cr refers to the ratio used in this manuscript to standardize measurements and correct for different urinary samples concentration/dilution as well as allow us to compare measurements overtime. The first MA/Cr measurement during the study period was considered the baseline, and the last measurement during the study period was utilized as the last MA/Cr. The rate of change in MA/Cr is expressed as micrograms per milligram per year. The study was approved by the MUSC Institutional Review Board.

Demographic and bioclinical variables collected from EMR and included in the current study include age, sex, urine MA, urine creatinine, total bilirubin, reticulocyte count and percentage, serum albumin, serum creatinine, hemoglobin, LDH, baseline hemoglobin electrophoresis results, transfusion history, hemochromatosis diagnosis, and medication use. For statistical analysis purposes and clinical plausibility, medications were grouped into four groups: ACEi/ARB, hydroxyurea, both ACEi/ARB and hydroxyurea, and other.

All samples collected during the study period were analyzed at the MUSC clinical laboratory using the same technology. Urinary albumin levels were measured using a solid-phase fluorescent immunoassay. Urinary creatinine levels were measured by using the Jaffe rate reaction with a CX3 analyzer (Beckman ASTRA, Brea, CA, USA).

### Statistical Analysis

Statistical tests of significance between MA/Cr categories were determined using Student’s *t*-test or chi square test for continuous and dichotomous variables, respectively. Participant demographic characteristics and laboratory measures were compared by change in MA/Cr (increase or decrease). Based on clinical plausibility and statistical significance, demographic and/or laboratory determinants were entered into the multivariate logistic regression and used to examine of change in MA classification (outcome variable). There was a significant interaction between age and the history of receiving at least one blood transfusion. To test the hypothesis that history of transfusion affected change in MA/Cr, a product interaction term was calculated by multiplying history of interaction (dichotomous) with continuous age and entered into the multivariate model. Data were analyzed using Stata (SE 13.1; College Station, TX, USA). A *p*-value of <0.05 was considered statistically significant.

## Results

Three hundred seventy-three children and young adults with sickle cell disease who had ≥2 MA/Cr measurements were included in this study. Age range was 1–22 years; mean 10.21 (5.23), 49.5% were males. Mean follow-up time was 3.12 ± 1.16 years. At baseline, 328 children had normal (<20 mg/L) MA level. Forty-five (12.1%) of children had MA (≥20 mg/L), of which 91% were ≥8 years and 21 (47%) continued to have MA at the end of the study period. During the study period, 24 new patients developed MA and 24 normalized their MA to levels <20 mg/L.

To study the direction and magnitude of change in MA over the study period, MA/Cr (micrograms per milligram) was calculated. The study population was categorized based on the direction of MA/Cr change over time (increase or decrease). Almost one-half (46%) of the study population (*n* = 174) had worsening of the MA/Cr levels with a median absolute change of 3 μg/mg (1, 11) and a mean rate of 20 μg/mg/year (Table 1). On the other hand, 54% (*n* = 199) had improvement in their MA/Cr with a median absolute change of −4 μg/mg (−10, −2) and a mean rate of 10 μg/mg/year.

**Table 1:** **Characteristics of study population by direction of MA/Cr change**.

	Urine MA/Cr decrease 53.35% (*n* = 199)	Urine MA/Cr increase 46.65% (*n* = 174)	Total *n* = 373	*p*-value
Time, years between first and last	3.13 (1.19)	3.12 (1.12)	3.12 (1.16)	0.93
Urine MA/Cr change median (25th, 75th ‰), µg/mg	−4 (−10, −2)	3 (1, 11)	−1 (−4, 2)	<0.001
Age, years (SD)	9.64 (5.16)	10.86 (5.25)	10.21 (5.23)	0.03
Sex (male), % (*n*)	48.91 (90)	51.09 (94)	49.46 (184)	0.10
Serum albumin, g/dL (SD)	4.09 (0.30)	4.09 (0.29)	4.09 (0.30)	0.97
Serum creatinine mg/dL (SD)	0.43 (0.18)	0.45 (0.17)	0.44 (0.18)	0.34
Hemoglobin, g/dL (SD)	9.34 (1.78)	9.34 (1.63)	9.34 (1.71)	0.98
Hemoglobin A2, % (SD)	3.67 (0.85)	3.68 (0.93)	3.67 (0.88)	0.97
Hemoglobin F, % (SD)	9.09 (7.99)	7.43 (7.17)	8.32 (7.65)	0.04
Hemoglobin S, % (SD)	67.01 (19.60)	63.37 (20.39)	65.31 (20.02)	0.08
Total bilirubin, mg/dL (SD)	2.55 (2.29)	2.26 (2.00)	2.42 (2.00)	0.16
First (baseline) MA/Cr, µg/mg	30 (103)	16 (57)	23 (85)	0.11
Last MA/Cr, µg/mg	11 (29)	100 (642)	53 (440)	0.05
Blood transfusion, yes % (*n*)	49.75 (99)	55.75 (97)	52.55 (196)	0.25
ACEi/ARB, yes % (*n*)	3.52 (7)	6.32 (11)	4.83 (18)	0.21
Hydroxyurea, yes % (*n*)	13.07 (26)	16.09 (28)	14.48 (54)	0.41
Both ACEi/ARB and hydroxyurea	2.51 (5)	2.30 (4)	2.41 (9)	1.00

Children who developed worsening MA/Cr were significantly more likely to be older (10.86 ± 5.25 years; *p* = 0.02) and have a lower Hb F but not higher Hb S.

There were no statistically significant differences in the reticulocyte count or percentage, albumin, serum creatinine, hemoglobin, hemoglobin A2, total bilirubin levels, or medication (ACEi/ARBs, hydroxyurea) use between the MA/Cr groups.

There was a significant interaction between age and the history of receiving at least one blood transfusion (*p* = 0.013). In multivariate logistic regression model stratified by history of transfusion, age and total bilirubin levels were predictive of worsening MA/Cr levels in patients who received at least one blood transfusion during the study period. Baseline MA level was not predictive of the change in MA/Cr (Table 2).

**Table 2:** **Predictors of worsening MA/Cr levels stratified by transfusion history during the study period**.

Variable	OR	*p*	95% conf. interval
**Received blood transfusion**			
Age	1.12	<0.01	1.06–1.19
Sex	1.35	0.32	0.74–2.46
Bilirubin	0.81	0.01	0.69–0.95
**No blood transfusion**			
Age	1.01	0.85	0.94–1.07
Sex	1.80	0.06	0.98–3.29
Bilirubin	0.86	0.20	0.69–1.08

*OR, odds ratio; stratified: p for interaction age × transfusion = 0.01*.

## Discussion

Renal injury starts early in life in patients with SCD. The kidney’s medullary environment is one of relative hypoxia, acidosis, and hypertonicity, all conditions that favor red blood cell sickling and hemoglobin S polymerization. It is thought that repeated vaso-occlusive events in the vasa recta lead to an ischemic–hyperperfusion-type injury, characterized pathologically by early glomerular enlargement followed by eventual focal segmental glomerulosclerosis, the most common cause of renal failure in SCD (1, 6, 15, 16).

As a consequence, younger patients with SCD have an increased glomerular filtration rate (GFR) and renal plasma flow, both of which normalize in adolescence and decrease in adulthood as a result of the scarring (17, 18). Proteinuria is the most common clinical manifestation of these changes of sickle nephropathy. Recently, a large number of studies have described the prevalence and correlates of microalbuminuria as an early marker of renal injury. To our knowledge, there are no longitudinal studies examining changes in MA levels in children with SCD. This is the first large pediatric study to assess the progression of MA levels (corrected to urinary creatinine levels) and to examine predictors of change over time in a cohort of patients with SCD. In this study, we report that MA/Cr levels can change both ways over time, almost one-half of the study population (*n* = 174) developed worsening MA/Cr. Older children were more likely to have MA (*p* = 0.03). Ninety-one percent of children with MA were 8 years of age or older. Age and bilirubin levels were predictive of MA/Cr level increase in children who received one or more blood transfusions during the study period.

Very little is known about MA rate of progression in children with SCD (8). Previously, we have reported the prevalence of MA in a pediatric cohort to be 15.5% (10). In the present study, the prevalence of MA was lower at 12.1% possibly due to the inclusion of younger children in the study cohort. Gosmanova and colleagues reported the prevalence and progression of MA and CKD over 5-year follow-up in 98 adults with SCD (19). In their study, the prevalence of abnormal albuminuria at baseline was 26.5% but subsequently increased to 42.8% after 5 years of follow-up. Most of the patients with abnormal albuminuria at baseline (65.4%) had unchanged, whereas a smaller number (11.5%) of patients progressed to a higher albuminuria grade, and 23.1% of patients regressed to a lower albuminuria grade. These findings are consistent with our findings that in a subset of children with SCD, MA may improve with time. This may reflect improved hemodynamic forces in the glomerulus, repair of tubular injury, resolution of systemic inflammation, ischemia reperfusion injury, or possibly withdrawal of offending medications. That being said, almost one-half of the study population developed worsening of the MA/Cr levels.

Other studies have defined MA as levels ≥30 μg/mg, using this cutoff level to reexamine our cohort, all (except 3) patients (*n* = 42) whom were diagnosed to have MA in our study had levels above ≥30 μg/mg. Of whom, 69% has worsening of their MA levels by the end of the study, and in 31%, MA levels improved although did not normalize. It is also important to point out that multiple studies examining different patient populations have demonstrated good correlation between spot urine MA/Cr and 24-h urinary albumin excretion in children (20).

While examining the predictors of MA/Cr progression, we found a significant interaction between age and blood transfusion history, which necessitated stratifying the statistical models by transfusion history. Children with history of one or more blood transfusion during the study period (probably sicker children with possibly more frequent crises, increased transcranial Doppler velocities, more severe anemia, and hemolysis requiring blood transfusion) were more likely to have worsening of MA/Cr if they were older and/or had higher bilirubin levels.

Among clinical correlates and predictors of MA that we and others have previously described are increasing age, lower hemoglobin levels, increased markers of hemolysis (10), such as reticulocyte count, indirect bilirubin level, and serum haptoglobin, and LDH levels (21). In this study, there were no statistically significant differences in the reticulocyte percentage or count, hemoglobin, hemoglobin A2, and bilirubin levels between the groups of worsening MA/Cr vs. improved MA/Cr levels. On the other hand, lower Hb F but not higher Hb S was associated with increasing MA/Cr levels; this is consistent with findings published by Lebensburger et al. showing protective role of fetal hemoglobin in early kidney disease for children with sickle cell anemia (22).

In children with SCD, age correlation to MA levels is well described, although the strength of the association varies (9, 11, 12, 23–25). In this study, age positively correlated with urinary albumin excretion (*p* = 0.03). The NHLBI Evidence-Based Management of Sickle Cell Disease Expert Panel Report, 2014, recommended screening all individuals with SCD, beginning by age 10, for proteinuria. If the result is negative, repeat screening annually. If the result is positive, perform first morning void urine albumin–creatinine ratio and if abnormal, consult with or refer to a renal specialist. For adults with MA without other apparent cause, the guidelines recommend to initiate angiotensin-converting enzyme (ACE) inhibitor therapy with moderate quality evidence (26). Although our study was not designed to determine a cutoff age for MA screening in children with SCD, 91% of children with MA were 8 years of age or older. Whether patients will benefit from screening at an earlier age using MA measurements or should clinician only screen with urinalysis or urine dipstick to avoid over diagnosing a condition (MA) that may improve with time remains to be answered.

Studies of therapeutic interventions, including ACE inhibitor and hydroxyurea therapy (HU), to reduce MA are limited, small, and retrospective. Although hydroxyurea treatment was associated with a decrease in hyperfiltration and LDH levels in the HUSTLE study, its effects on cystatin C and MA were not significant (27). A more recent study in 58 adults with SCD found 6 months of hydroxyurea treatment to reduce MA levels (28). On the other hand, in general, studies examining ACEi and ARB treatment demonstrated reduction in MA levels, keeping in mind those studies were limited by small sample size and duration of follow-up (8, 9, 16, 29, 30). A small proportion of our cohort received hydroxyurea or ACEi/ARB or both treatments during the study period; the number of subjects who received these treatments was comparable between the MA increase vs. MA decrease groups (Table 1). While short trials of ACEi showed beneficial effects on reduction of MA levels, using such treatments for extended periods of time raise multiple questions. For example, are the effects of such treatment long-lived or transient? Does reduction of MA levels translate to reduction in long-term risk of ESRD or CKD progression? Do such treatments raise the risk of AKI and/or hyperkalemia in this “at risk” patient population? How big of an impact do these agents have on blood pressure in children with SCD? A large prospective study designed and powered to examine the safety and efficacy of long-term treatment with these agents is needed (9, 16, 30–32).

Our study is limited by the retrospective study design utilizing data from the EMR. The data set did not have certain sickle cell comorbidity diagnosis (number of acute chest syndrome and other crises hospitalizations, history of stroke, or abnormal transcranial Doppler velocities); such comorbidities reflect to a certain degree the disease severity. To help assess for disease severity, variables, such as markers of hemolysis and history of blood transfusion, were included in our analysis. Our data set did not have growth and nutrition (anthropometric data) and cardiovascular parameters, variables that should be included in future prospective studies. We relied on ICD-9 codes to identify patients with sickle cell disease from our EMR, this possibly introduced errors related to coding. Our clinic staff was instructed to collect midstream first urine samples, the fact that this is a retrospective study using EMR data carries the possibility that patients (children) scheduled for clinic visits later in the day may have provided samples otherwise. Our study has multiple strengths, including the availability of longitudinal data on a relatively large cohort and the availability of both MA and urinary creatinine levels, which allowed us to accurately compare levels over the study period. In addition, all samples were collected analyzed at the one institution and using the same equipment.

Urinary microalbumin levels are considered to be one of the early renal injury markers in patients with sickle cell disease. Testing for it is non-invasive, readily available, and easy to perform in most of the clinical settings. Our study suggests that in children with SCD, MA levels may increase or decrease over time. The relationship between MA and SCD is a complex one with multiple cardiovascular and anthropometric confounding factors. Understanding the prevalence, significance, and the natural history of MA as well as identifying the subset of patients with SCD who are at risk of disease progression will allow us to target this high risk group with potential therapeutic interventions aiming at early renal protection.

## Glossary

Microalbuminuria (MA): urinary albumin measuring 20 mg/L or more on spot urine sample.

MA/Cr: microalbumin-to-creatinine ratio measured in micrograms of albumin to milligrams of creatinine.

## Author Contributions

IS, ML, SH, and SQ contributed to study design, IRB and protocol approval, interpretation of results, and drafting the manuscript. SH contributed to statistical analysis, interpretation of results, and drafting the manuscript.

## Conflict of Interest Statement

The authors declare that the research was conducted in the absence of any commercial or financial relationships that could be construed as a potential conflict of interest.
